# Activation of GPR40 induces hypothalamic neurogenesis through p38- and BDNF-dependent mechanisms

**DOI:** 10.1038/s41598-020-68110-2

**Published:** 2020-07-06

**Authors:** Daiane F. Engel, Vanessa C. D. Bobbo, Carina S. Solon, Guilherme A. Nogueira, Alexandre Moura-Assis, Natalia F. Mendes, Ariane M. Zanesco, Athanasios Papangelis, Trond Ulven, Licio A. Velloso

**Affiliations:** 1 Laboratory of Cell Signaling and Obesity and Comorbidities Research Center, University of Campinas, Campinas, SP 13084-970 Brazil; 2 Department of Drug Design and Pharmacology, University of Copenhagen, 2100 Copenhagen, Denmark

**Keywords:** Neurochemistry, Adult neurogenesis

## Abstract

Hypothalamic adult neurogenesis provides the basis for renewal of neurons involved in the regulation of whole-body energy status. In addition to hormones, cytokines and growth factors, components of the diet, particularly fatty acids, have been shown to stimulate hypothalamic neurogenesis; however, the mechanisms behind this action are unknown. Here, we hypothesized that GPR40 (FFAR1), the receptor for medium and long chain unsaturated fatty acids, could mediate at least part of the neurogenic activity in the hypothalamus. We show that a GPR40 ligand increased hypothalamic cell proliferation and survival in adult mice. In postnatal generated neurospheres, acting in synergy with brain-derived neurotrophic factor (BDNF) and interleukin 6, GPR40 activation increased the expression of doublecortin during the early differentiation phase and of the mature neuronal marker, microtubule-associated protein 2 (MAP2), during the late differentiation phase. In Neuro-2a proliferative cell-line GPR40 activation increased BDNF expression and p38 activation. The chemical inhibition of p38 abolished GPR40 effect in inducing neurogenesis markers in neurospheres, whereas BDNF immunoneutralization inhibited GPR40-induced cell proliferation in the hypothalamus of adult mice. Thus, GPR40 acts through p38 and BDNF to induce hypothalamic neurogenesis. This study provides mechanistic advance in the understating of how a fatty acid receptor regulates adult hypothalamic neurogenesis.

## Introduction

Neurons of the mediobasal hypothalamus play central roles in the homeostatic control of food intake and energy expenditure^1,2^. They are responsive to hormones, neural signals, and nutrients that indicate the energy stores in the body; as long as orexigenic and anorexigenic responses are preserved, body mass stability is sustained over time^3–5^. However, a number of environmental and genetic factors can affect the function and viability of hypothalamic neurons, changes that result in abnormal regulation of body mass^6–9^.

In aging and obesity, hypothalamic neurons are damaged by inflammation; they undergo abnormal function and eventually apoptosis^10–15^. This phenomenon generates an imbalance in neuronal orexigenic and anorexigenic subpopulations and further contributes to the progression of increased adiposity and metabolic complications^10–15^. If neuronal loss is prevented by modifications in the diet or inhibition of hypothalamic inflammation, whole body energy homeostasis is restored^8,15^. However, upon long-lasting exposure to the damaging effects of obesity and aging, neuronal loss may be reverted only by the generation of new neurons^16,17^.

Physiological adult hypothalamic neurogenesis occurs at a much lower rate than neurogenesis in the subventricular and subgranular zones, the most important anatomical sources of newborn neurons in adulthood^18^. Nevertheless, under certain types of stimuli, hypothalamic neurogenesis can increase substantially and impact whole body energy homeostasis^9,12,17^. This phenomenon occurs for stimuli provided by growth factors, such as ciliary neurotrophic factor (CNTF), fibroblast growth factor 10 (FGF10), and brain-derived neurotrophic factor (BDNF)^17,19,20^, as well as hormones, such as leptin, insulin, and estradiol^12,21,22^.

Diet can also modulate hypothalamic neurogenesis; as compared to solid diet, liquid diet results in decreased cell proliferation in the hypothalamus of adult rodents^23^. Nutrients can impact either positively or negatively the generation of new neurons depending on the type of nutrient that is employed^12,24^. Mono- and polyunsaturated fatty acids (MUFAs and PUFAs, respectively) act through distinct pathways in the hypothalamus by controlling food intake, energy expenditure, systemic glucose metabolism, and inflammation^25–29^. One of the putative mechanisms behind the beneficial effects of MUFAs and PUFAs in the hypothalamus is neurogenesis^30,31^. We have previously shown that one PUFA, docosahexaenoic acid (DHA), induces hypothalamic neurogenesis via the activation of medium- and long-chain fatty acid receptor, GPR40^30^, which is also regarded as a mediator of the neurogenic effect of PUFAs in the hippocampus^32^. However, the mechanisms that drive GPR40-dependent induction of hypothalamic neurogenesis are currently unknown. Here, employing potent synthetic agonists of GPR40 in living animals, neuronal cell culture, and neurospheres, we demonstrated that BDNF and p38 are important components of the system mediating GPR40-induced hypothalamic neurogenesis.

## Results

### GPR40 modulated proliferation and survival of hypothalamic adult neural precursor cells

**Figure 1:**
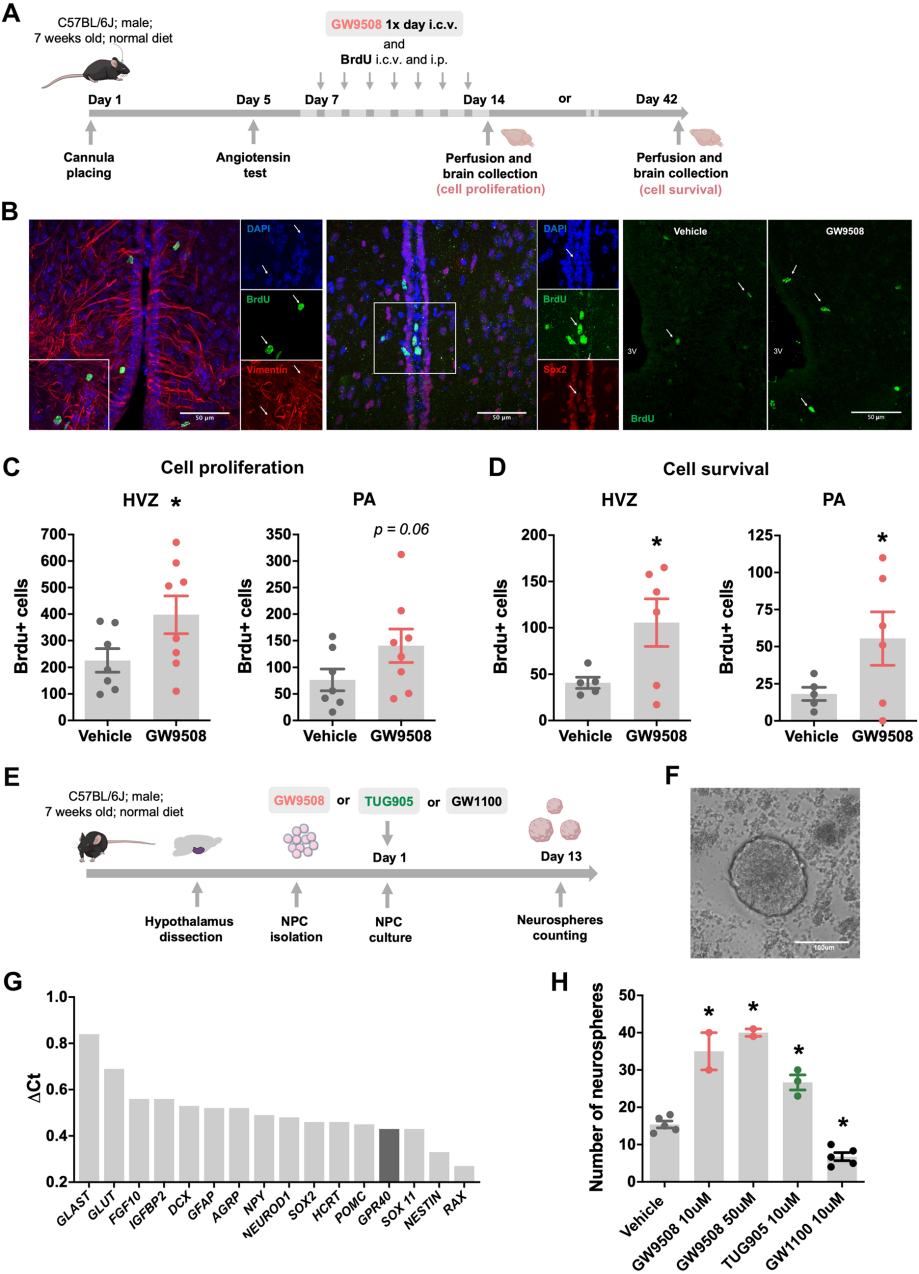
GPR40 modulates cell proliferation and survival in the hypothalamus of adult mice. C57BL/6J mice received a 7-day repeated treatment of GW9508 or vehicle, and BrdU, were sacrificed 24 h or 28 days after the last BrdU injection by transcardial perfusion and their brains were processed for immunohistochemistry (**A**). The co-labeling of BrdU/vimentin and BrdU/sox2 positive cells indicates the neural precursor phenotype of newborn cells after 24 h of BrdU injections (**B**). Panel **B** also shows representative images of BrdU-positive cells in the hypothalamic ventricular zone (HVZ) of vehicle and GW9508 treated mice after 24 h. The GW9508 treated mice showed increased number of BrdU immunopositive cells in the in the HVZ (**C**). Immunolabeling for BrdU-positive cells present in the hypothalamus 28 days after the last BrdU administration reveals higher survival of newborn cells in both the HVZ and parenchyma (PA) of GW9508 treated mice (**D**). White arrows indicate either BrdU, vimentin and sox2 immunopositive cells in the HVZ. Scale bars = 50 μm (**B**). The effect of GPR40 over adult NPC proliferation was also assessed ex vivo. Cell proliferation was estimated by quantifying the number of primary neurospheres generated after 13 days exposure to GPR40 agonists (GW9508 and TUG905) and antagonist (GW1100) (**E**). Phase-contrast image of hypothalamic neurosphere generated from adult hypothalamic NPC cells and cultured with growth factors in non-adhesive conditions (**F**). Neurospheres obtained from control group showed high mRNA expression of NPC markers and hypothalamus-related genes (**G**). GPR40 activation increased the number of generated neurospheres, while its inhibition reduced NPC proliferation (**H**). Scale bar = 100 μm (**F**). Data are presented as means ± SEM. N = 5–7 per group (**C**, **D**) 1 (**G**) and 2–5 preparations (**H**). *p < 0.05, *t*-test (**C**, **D**); one-way ANOVA followed by Tukey’s post hoc test (**H**).

To address the putative involvement of GPR40 activation on adult hypothalamic neurogenesis, we determined the rates of cellular proliferation in 8-week old C57BL/6J mice treated with GW9508 or vehicle. Both experimental groups were injected via intracerebroventricular (icv) and intraperitoneal (ip) routes with the thymidine analogue 5-bromo-2′-deoxyuridine (BrdU) and euthanized 24 h or 28 days later (Fig. 1A). The phenotype of BrdU-positive cells was characterized by colocalization with the neural precursor cell markers Sox2 and vimentin (Fig. 1B). As shown in Fig. 1C, in the hypothalamic ventricular zone (HVZ), GW9508-treated mice presented increased numbers of BrdU-labeled cells compared to wild-type mice, data that indicate increased proliferation of precursor cells. In addition, in order to estimate cell survival, we determined the number of newly generated cells that persisted four weeks after BrdU incorporation; as shown in Fig. 1D, there was an increase in the number of BrdU-labeled cells in the GW9508 treated animals in both the HVZ and parenchyma (PA).

Next, we generated primary neurospheres from the hypothalami of adult mice (Fig. 1F). This assay provides a good read-out for the effects of extrinsic factors on proliferative and differentiation potential of neural precursor cells (NPC). After 13 days in culture, the neurospheres showed high messenger RNA (mRNA) expression of NPC markers and other hypothalamus-related genes (Fig. 1G). Validating the results obtained in living mice, isolated cells exposed to GPR40 agonists, GW9508 or TUG905, presented increased cell proliferation and, consequently, a greater number of neurospheres. Conversely, GPR40 signaling inhibition, via the antagonist GW1100, reduced the number of neurospheres (Fig. 1H).

### Activation of GPR40 in synergy with BDNF and interleukin-6 (IL-6) increased neuronal differentiation of hypothalamic neural precursor cells

**Figure 2:**
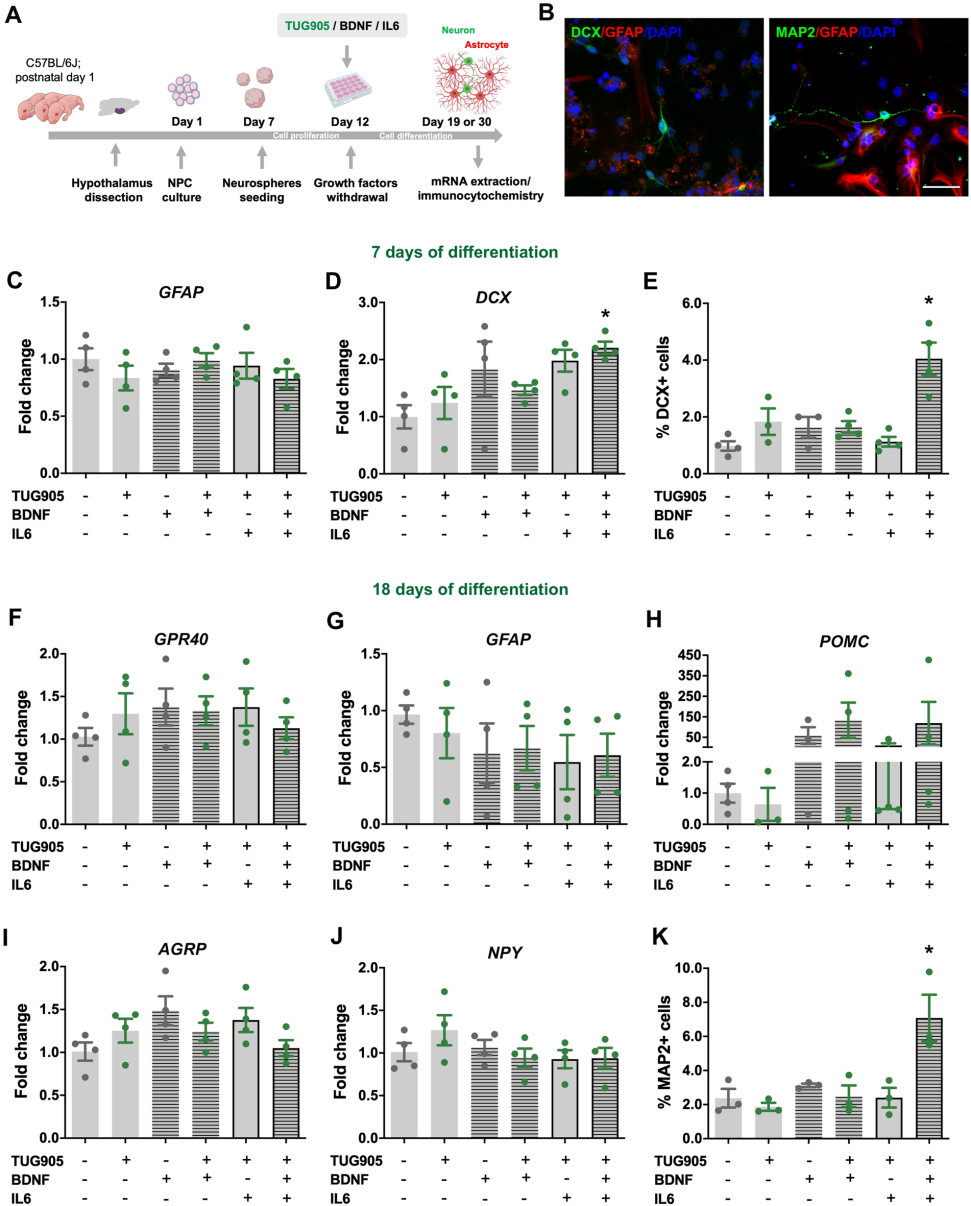
Activation of GPR40 in synergy with BDNF and interleukin-6 increases neuronal differentiation of hypothalamic neural precursor cells. Neurospheres were prepared from hypothalamic NPC of postnatal day 1 C57BL/6J mice and treated with TUG905, BDNF or Il-6 during 7 or 18 days of differentiation (**A**). Immunostaining of DCX/GFAP positive cells in 7 days differentiated cultures and of MAP2/GFAP positive cells in 18 days differentiated cultures (**B**). Gene expression analysis of 7 days differentiated NPC (**C**, **D**) identified increased mRNA levels of DCX in TUG905, BDNF and Il-6 treated cells (**D**) as well as a higher number of DCX-positive neuroblasts (**E**). In 18 days differentiated NPC there was no difference on gene expression of GPR40, GFAP or hypothalamic neuropeptides after TUG905, BDNF or Il-6 treatments (**F**–**J**), despite the increase on MAP2-positive mature neurons (**K**). Scale bar 25 μm. Data are presented as means ± SEM. N = 3–4 per group per group. *p < 0.05, one-way ANOVA followed by Tukey’s post hoc test.

To investigate the effect of GPR40 on NPC differentiation, we generated neurospheres from mouse hypothalamic tissue obtained at postnatal day 1. Neurospheres were allowed to proliferate in monolayers and then induced to differentiate upon TUG905, BDNF, and IL-6 treatments, individually or in combination, for 7 or 18 days (Fig. 2A). DCX/GFAP positive cells were detected in cultures differentiated for 7 days and MAP2/GFAP positive cells in cultures differentiated for 18 days (Fig. 2B). At day 7, the combination of TUG905, BDNF, and IL-6 increased mRNA levels of the neuroblast marker, DCX (Fig. 2D), as well as the number of DCX-immunopositive cells (Fig. 2E). At day 18, hypothalamic NPC differentiated into functional neurons that expressed feeding-related neuropeptides NPY, AgRP, and POMC^33^. Although we did not find any differences in the mRNA levels of these particular neuropeptides, in synergy with BDNF and IL6, GPR40 activation resulted in an increased number of mature neurons (MAP2-immunopositve cells; Fig. 2H).

### GPR40 signaling induced BDNF expression and p38 phosphorylation in Neuro2a proliferative cells

**Figure 3:**
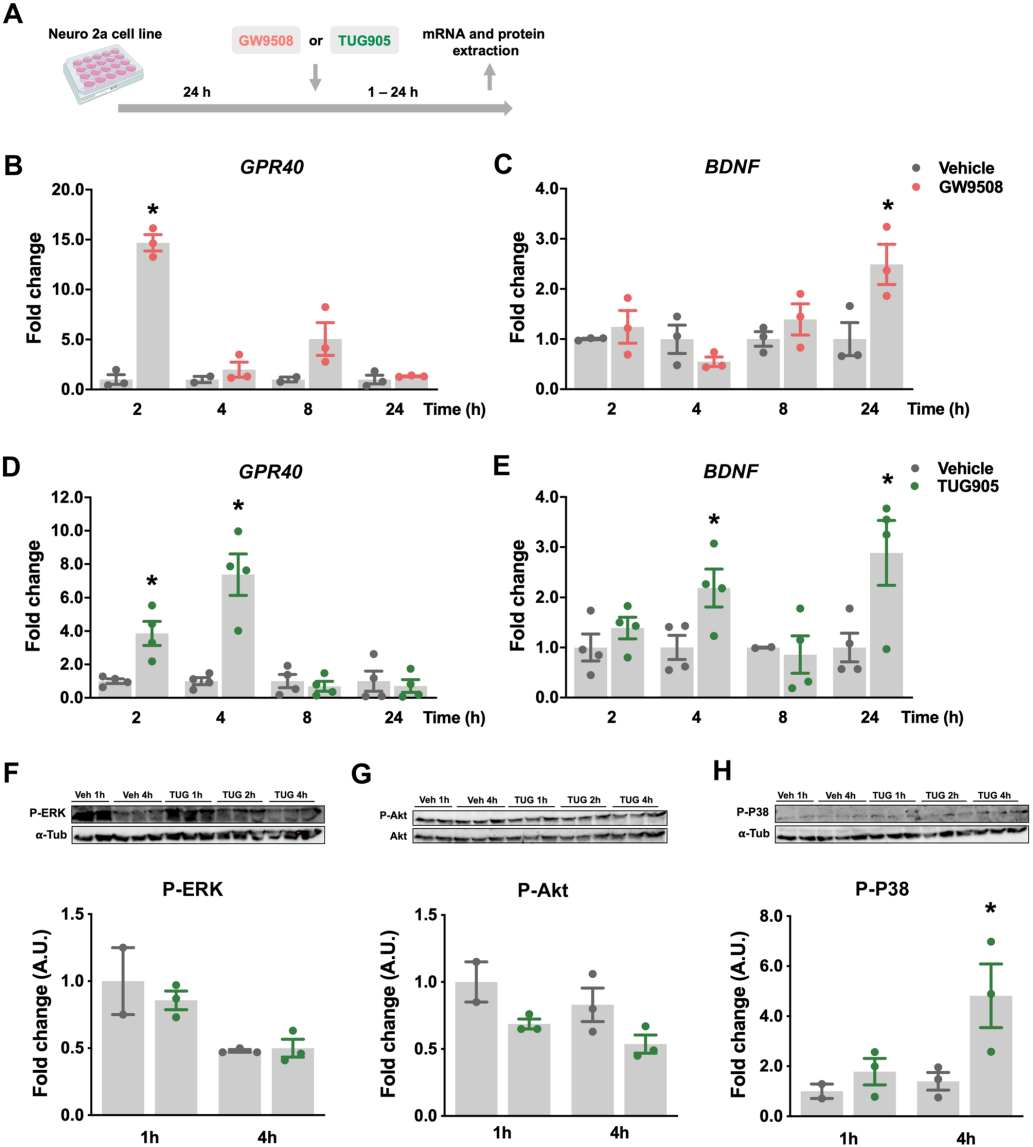
GPR40 signaling induces BDNF expression and p38 phosphorylation in Neuro2a proliferative cells. GPR40 and BNDF transcripts and phosphorylation of ERK, Akt and P38 proteins were measured after GW9508 and TUG905 treatment of Neuro2a cells (**A**). GW9508 treatment induced GPR40 transcript after 2 h (**B**) and BDNF after 24 h (**C**), while TUG905 increased GPR40 gene expression in 2 and 4 h (**D**) and BDNF after 4 and 24 h (**E**). TUG905 did not change ERK and Akt phosphorylation (**F**, **G**) but increased p38 phosphorylation after 4 h (**H**). Membranes were reblotted with antibodies against alpha-tubulin (α-Tub; F and H) and total Akt (**G**). Data are presented as means ± SEM. N = 2–3 (**B**, **C**; **F**–**H**) and 4 (**D**, **E**) per group. *p < 0.05, *t*-test (**B**–**E**); one-way ANOVA followed by Tukey’s post hoc test (**F**–**H**).

In order to evaluate the signaling pathways involved in GPR40 activation, Neuro2a (murine neuroblastoma) cells were treated with GPR40 synthetic ligands for 1–24 h (Fig. 3A). The time-course assay revealed that GW9508 increased GPR40 gene expression after 2 h (Fig. 3B), while BNDF mRNA levels were increased after 24 h (Fig. 3C). A selective ligand, TUG905, increased GPR40 gene expression 2 and 4 h after treatment; it also increased BDNF expression at 4 and 24 h of treatment (Fig. 3D, E).

To determine the potential involvement of extracellular-signal-regulated kinase (ERK), Akt, and p38 signaling pathways in the intracellular response to GPR40 activation, we employed immunoblotting to determine protein-specific phosphorylation at 1 and 4 h after TUG905 treatment. We found an increased phosphorylated p38 expression 4 h after TUG905 treatment, with no effects on other proteins, in these experimental conditions (Fig. 3F, G).

### P38 and BDNF were implicated in the GPR40 effect on hypothalamic neural precursor cells

**Figure 4:**
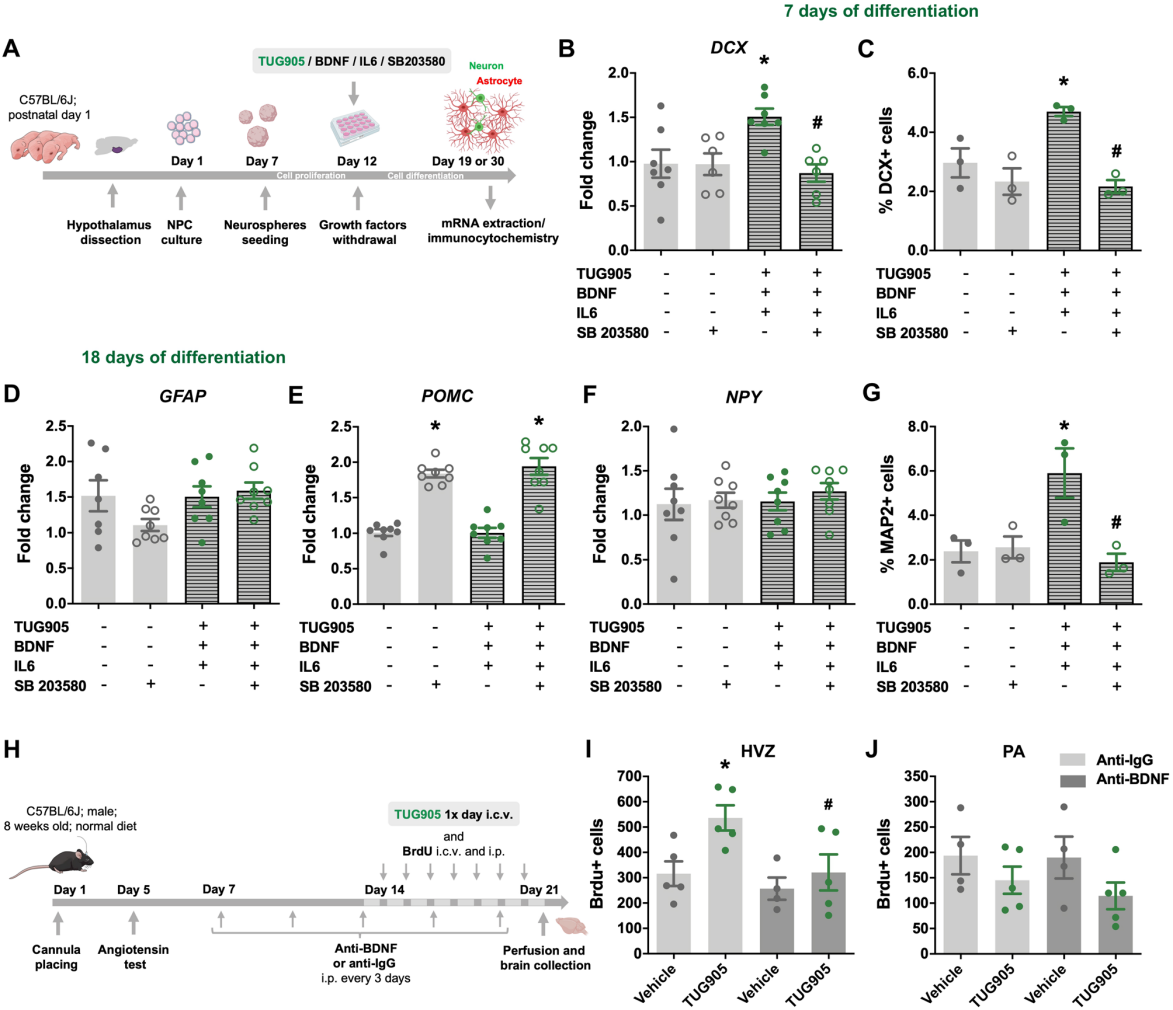
P38 and BDNF are implicated in GPR40 effect on hypothalamic NPC. Neurospheres were prepared from hypothalamic NPC of postnatal day 1 C57BL/6J mice and treated with TUG905, BDNF, Il-6 or p38 inhibitor (SB203580) during 7 or 18 days of differentiation (**A**). Gene expression analysis of 7 days differentiated NPC (**C**, **D**) demonstrated that SB203580 prevented the effect of TUG905, BDNF and Il-6 combined treatment in increasing DCX mRNA (**B**) and immunostaining (**C**). In 18 days differentiated NPC there was an effect of SB203580 in POMC mRNA levels with no changes in GFAP or NPY transcript (**D**–**F**). The increase in MAP2-positive mature neurons produced by TUG905, BDNF and Il-6 combined treatment was also abolished by p38 inhibition (**G**). To assess the involvement of BDNF in the GPR40 effect over adult hypothalamic NPC proliferation, C57BL/6J mice received, every 3 days, an immunoneutralizing rabbit anti-BDNF antibody or control IgG for 14 days, before and during the 7-day repeated treatment with TUG905 or vehicle, and BrdU. Right after treatments, mice were sacrificed by transcardial perfusion and their brains were processed for immunohistochemistry (**H**). The TUG905 treated mice showed increased number of BrdU immunopositive cells in the in the hypothalamic ventricular zone (HVZ), which was abolished by anti-BDNF administration (**I**), and no effect in the hypothalamic parenchyma (PA) (**J**). Data are presented as means ± SEM. N = 6–8 (B;D-F), 3 (**C**;**G**) and 4–6 (**I**, **J**) per group. *p < 0.05, one-way ANOVA followed by Tukey’s post hoc test.

Given that TUG905 increased the activation of p38, an important protein that mediates neurogenesis^34^, we tested whether p38 inhibition could disturb the neurogenic effect of TUG905/BDNF/IL-6 combined treatment in postnatal hypothalamic NPC (Fig. 4A). The p38 inhibitor SB203580 abolished the effect of TUG905/BDNF/IL-6 to increase DCX gene and protein expression in 7-day differentiated cells (Fig. 4B and C). In concert, after 18 days of differentiation, the increase in the number of MAP2-positive mature neurons produced by the combined treatment was abolished when cells were exposed to the inhibitor (Fig. 4G).

BDNF is an important player in adult hypothalamic NPC proliferation^20^. Considering that its expression is induced upon GPR40 activation, we asked whether BDNF immunoneutralization would affect GPR40-induced cell proliferation in the hypothalamus of adult mice (Fig. 4H). As for GW9508 (Fig. 1), central TUG905 injection induced an increase in NPC proliferation in the HVZ, an effect that was abolished by treatment with anti-BDNF antibody (Fig. 4I).

## Discussion

Nutrients are undisputedly among the most relevant environmental factors that impact health and disease^35^. Great efforts have been put into the characterization of the effects of Mediterranean and Innuit diets in metabolic and cardiovascular diseases^36,37^, dietary salt content in hypertension^38,39^, and fructose in diabetes^40,41^, only to cite some examples of unquestionable associations. In neurodegenerative diseases, particularly Alzheimer’s disease, studies have suggested that dietary components might play important roles in the prevention of disease development and attenuation of patients’ symptoms^42,43^. There is considerable evidence for the beneficial roles of MUFAs and PUFAs for preventing and attenuating the course of progressive memory impairment^44^, and the increase in the neurogenic capacity has been proposed as one of the mechanisms behind those actions^45,46^.

In the hypothalamus, neuronal decay associated with obesity and aging results from molecular and cellular abnormalities that are similar to the ones that occur in the hippocampus in Alzheimer’s disease^16,47^. Likewise, MUFAs and PUFAs can exert beneficial effects in the hypothalamus by reducing inflammation and restoring whole body energy balance and glucose tolerance^25,27^. There are a number of mechanisms involved in the hypothalamic responses to MUFAs and PUFAs^25–29^, including increased neurogenesis^30^. We have previously shown that the neurogenic effects of DHA in the hypothalamus are mediated by GPR40; however, the mechanisms involved in this response were previously unknown.

In order to explore these mechanisms, we employed two chemical ligands that potently activate GPR40: GW9508 and TUG905. GW9508 is a commercially available agonist that was initially regarded as specific for GPR40 (pEC_50_ = 7.3); however, further studies have demonstrated that it can also activate GPR120^48,49^. TUG905 is synthetized by us, and it is undisputedly specific for both human GPR40 (pEC_50_ = 8.1)^50^ and the mouse orthologue (pEC_50_ = 7.0)^51^. In adult mice, GW9508 increased hypothalamic cell proliferation and survival, and at least a portion of BrdU-positive cells colocalized with neural precursor cell markers, namely Sox2 and vimentin, thus representing a neurogenic stimulus^17^. Furthermore, in NPC prepared from dissected adult hypothalamus, GW9508 and TUG905 increased adult neurosphere proliferation, whereas GPR40 inhibition with the potent synthetic inhibitor GW1100 significantly inhibited proliferation. These data reproduced findings of our previous study and reinforced the neurogenic action of GPR40^30^. Moreover, because inhibition of GPR40 in neurospheres resulted in proliferation levels below baseline, we suggest that GPR40 is involved in steady-state maintenance of neurogenic activity.

Neurogenesis results from the synergistic actions of multiple stimuli^52^. An important stimulus, acting in the hypothalamus as well as other neurogenic niches, is BDNF^53^. During neonatal neuron development, there is a surge in the expression levels of BDNF and its receptor involved in neurogenesis (TrkB). Further, the genetic ablation of BDNF severely impairs neurogenic activity^54,55^. Conversely, either the exogenous injection or the overexpression of BDNF in neurogenic niches promotes a substantial increase in neurogenesis^56,57^. IL-6 is another endogenous substance known to promote adult neurogenesis^58^. With regard to BDNF, IL-6 undergoes a rapid and transient increase during the perinatal period, a phenomenon that is related to neurogenic activity^59^. In addition, exogenous IL-6 promotes adult neurogenesis, whereas the genetic ablation of the IL-6 receptor impairs the development of neural stem cells^59^. Thus, in order to further explore the neurogenic activity of GPR40, we performed experiments using a combination of the GPR40 specific agonist, TUG905, with BDNF and IL-6. In neurospheres, the specific activation of GPR40 acted in synergy with BDNF and IL-6 to increase the expression of DCX and the mature neuron marker MAP2. Moreover, in a proliferative neuronal cell-line, TUG905 increased BDNF expression, which is consistent with the effect of DHA acting directly in the hypothalamus of adult mice^30^.

Next, we evaluated three signaling systems that are potently involved in the transduction of the GPR40 signal in hypothalamic neurons: ERK, Akt, and p38. All these signaling systems have been previously described as involved in different aspects of adult neurogenic activity^34,60,61^. TUG905 significantly increased the phosphorylation of p38; however, it did not modify the molecular activity status of ERK and Akt. The MAPK family member p38 is highly expressed in the brain, and studies have demonstrated its crucial role in cell proliferation and differentiation^62,63^. In the hippocampus, p38 deficiency results in impaired long term memory, a phenomenon that may be partially due to its involvement in neurogenesis^64,65^.

**Figure 5:**
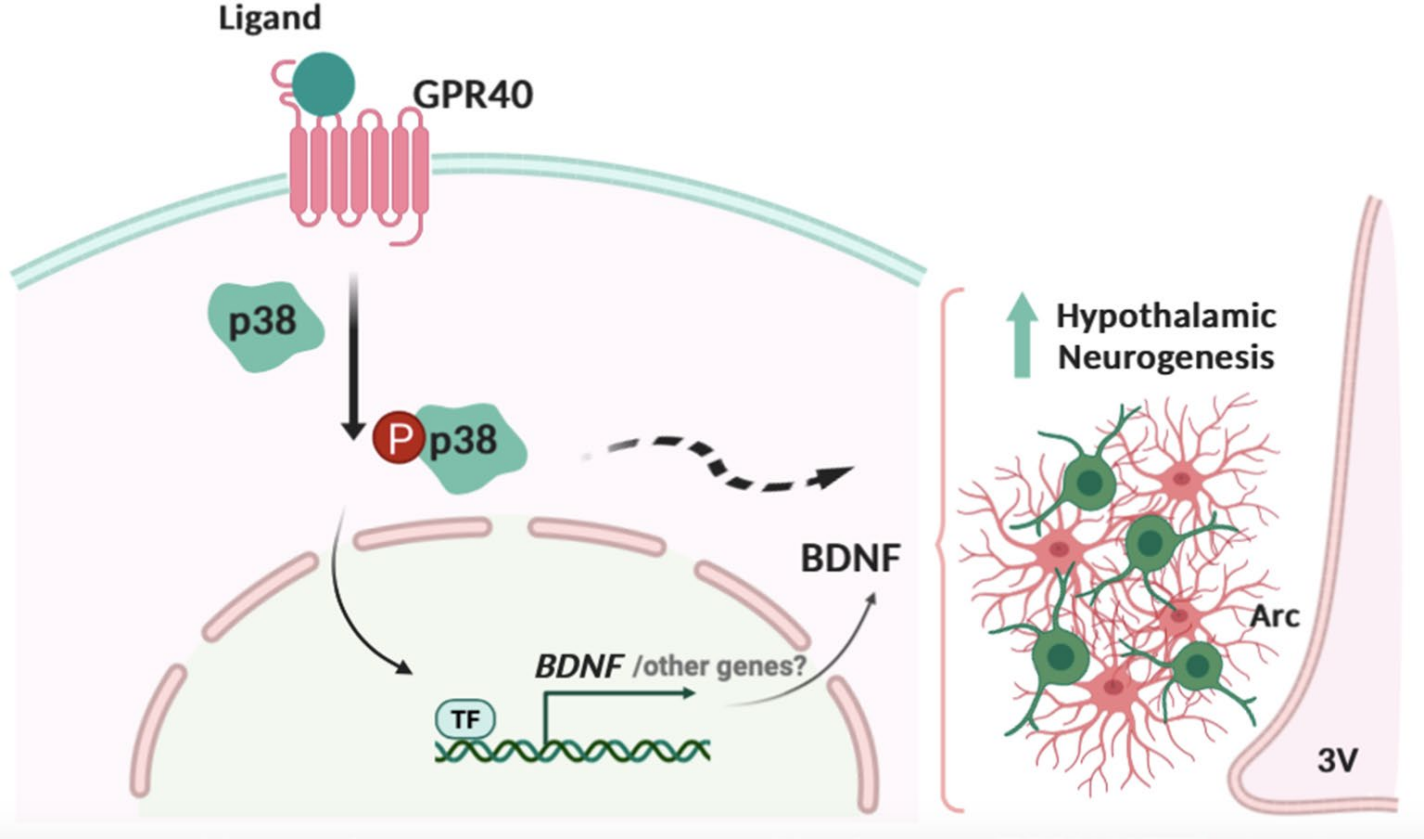
Proposed mechanism for GPR40 influence in hypothalamic neurogenesis. The synthetic GPR40 ligands increase P38 phosphorylation and BDNF gene expression. Disruption of P38/BDNF signaling impaired the response on hypothalamic neural precursor cells proliferation and differentiation, indicating its involvement in the mechanism of GPR40-induced hypothalamic neurogenesis. *3V* third ventricle, *Arc* arcuate nucleus, *BDNF* brain derived neurotrophic factor, *GPR40* G protein coupled receptor 40 or free fatty acid receptor 1, *P38* P38 mitogen-activated protein kinase, *TF* unknown transcription factor.

In the final part of the study, we used distinct approaches to inhibit either p38 or BDNF and determine their involvement in GPR40-induced activation of hypothalamic neurogenesis. In neurospheres, p38 inhibition completely suppressed the synergistic effects of TUG905, BDNF, and IL-6 to induce DCX and MAP2. In adult mice, BDNF immunoneutralization inhibited the proliferative action of TUG905 in the hypothalamus. Taken together, these results provide evidence for the important roles of BDNF and p38 signaling in the GPR40-dependent induction of neurogenesis in the hypothalamus. Previous studies demonstrated that p38/BDNF coupled signaling mediates neurite outgrowth and neuronal survival in vitro^66^, hippocampal neuronal synaptic development^67^ and neuroprotection upon induced ischemia^68^. In the hippocampus BDNF synthesis requires p38 and involves N-type voltage-gated calcium channels and/or adenosine A1 receptors activation^69^. However, this is the first study demonstrating p38 and BDNF combined action in mediating adult hypothalamic neurogenesis (Fig. 5).

In conclusion, we advanced the characterization of the mechanisms mediating GPR40-induced hypothalamic neurogenesis. These data reinforce the role of unsaturated fatty acids in hypothalamic neuronal fitness and expand the window of opportunity for drug development aimed at restoring hypothalamic activity in aging, obesity, and metabolic diseases.

## Methods

### Experimental animals

Male C57BL/6 mice were obtained from the Animal Facility of the University of Campinas, originally purchased from Jackson Laboratory (JAX stock #000,664). All mice were kept in individual cages at 21 ± 5 °C, in 12/12 h light/dark cycle, with water and chow available ad libitum. Animals were housed in groups of five or individually, when submitted to central cannula implantation. In all experiments, control and intervention group mice were submitted to the same experimental settings. All experiments were conducted according to the "Guide for the Care and Use of Laboratory Animals of the Institute of Laboratory Animal Resources, US National Academy of Sciences" and were approved by the Ethics Committee (Comissão de Ética no Uso de Animais/Instituto de Biologia /Universidade Estadual de Campinas no. 4,948-1/2018).

### In vivo experiments

#### Experimental protocol

For central administration of GPR40 exogenous ligands, 7-week old mice were submitted to cannula implantation in the right lateral ventricle under xylazine (10 mg/kg, ip) and ketamine (100 mg/kg, ip) anesthesia. The coordinates were as follows: anteroposterior, 0.34 mm; lateral, 1.0 mm; and depth, 2.2 mm. The efficiency of cannula placement and viability was confirmed by icv administration of angiotensin II and measurement of the drinking response. Ventricular-cannulated mice were treated daily for 7 days with 2.0 μL of vehicle [1:1:3; Ethanol/ DMSO/ artificial cerebrospinal fluid (Tocris)], GW9508 (2.0 mM; Tocris Bioscience) or TUG905 (2.0 mM, synthesized as previously described^50^.

BrdU (Sigma) was used to evaluate cell proliferation and survival. BrdU is a thymidine analogue that is incorporated into the DNA double-helix during the S-phase of the cell cycle, and thus marks actively proliferating cells^70^. All animals received BrdU (0.1 M phosphate-buffered saline [PBS], pH = 7.2; 10 μg/day icv and 50 mg 1 × day ip) and were euthanized 1 or 28 days later (Fig. 1A) to assay proliferation or survival of new cells, respectively.

For some experiments, mice were treated either with an immunoneutralizing rabbit anti-BDNF antibody (0.8 μg/100 μL, sc-546, Santa Cruz Biotechnology) or a pre-immune rabbit serum (R9133, Sigma), by ip injection every 3 days for 2 weeks.

#### Immunofluorescence staining

Mice were deeply anesthetized with a solution of xylazine (10 mg/kg, ip) and ketamine (100 mg/kg, ip) and perfused through the left cardiac ventricle with 0.9% saline solution, followed by 4% paraformaldehyde (PFA) in 0.1 M PBS (pH 7.4). After perfusion, the brains were removed, post-fixed in the same fixative solution for 24 h at room temperature (RT), and immersed in a 30% sucrose solution in PBS at 4 °C. Serial coronal Sects. (20 μm) of hypothalami were obtained with a cryostat (LEICA Microsystems, CM1860).

To analyze cell proliferation and survival in the HVZ and PA, a series of one-in-six free-floating sections were processed for detection of the BrdU immunoreactivity. The neural progenitor phenotype was assessed by double labeling BrdU/vimentin and BrdU/sox2 (proliferation group). Briefly, after DNA denaturation in 2 N HCl at room temperature (RT) for 1 h and pre-incubation with 10% blocking solution (0.1 M PBS with 10% normal goat or donkey serum and 0.2% Triton X-100), sections were incubated overnight at 4 °C in rat anti-BrdU (1:200; Ab6326), mouse anti-vimentin (1:200; sc-373717) and goat anti-sox2 (1:200; sc17320) primary antibodies. The sections were then incubated with secondary antibodies goat anti-rat FITC (1:200; sc2011), donkey anti-rat Alexa 488 (1:500; ab150153), goat anti-mouse rhodamine (1:200; sc2300) and donkey anti-goat Cy3 (1:500; ab6949) for 2 h at RT. All sections were mounted, cover slipped with Fluor Mount (Sigma), and stored at 4 °C.

The morphological analyses were performed on coded slides, with the executing researcher blinded to the experimental group. The total numbers of BrdU-immunopositive cells in the HVZ and PA were estimated by manually counting all positive cells. From all sections containing the hypothalamus from 1.06 to 2.30 mm posterior to Bregma, in one in-six series of sections were used for the analysis. The results were expressed as the total number of labeled cells by multiplying average number of labeled cells/sections by the total number of 20 μm thick sections (estimated as 62 sections).

Double-labeling was confirmed by three-dimensional orthogonal reconstruction (Imaje J software) of z-series of confocal microscopy covering the entire nucleus (or cell) of interest (confocal microscope Upright LSM780-NLO). The representative images are the stack of the z-project of all images obtained within the 20 μm section.

### Ex vivo experiments

#### Adult neurosphere culture

Seven-week old mice were euthanized, the brains were immediately removed, and the hypothalami microdissected. The tissue was minced with a scalpel blade, following a 0.05% trypsin-ethylenediaminetetraacetic acid (EDTA) digestion as described for subventricular zone neurosphere preparation^71^. The final pellet was resuspended in 1 mL of neurosphere growth medium/hypothalamus: Neurobasal medium (Gibco, Life Technologies) supplemented with 2% B27 (Invitrogen), 500 mM/L-Glutamine (Life Technologies), 50 units/mL penicillin/streptomycin (Life Technologies), 20 ng/mL human basic fibroblast growth factor (bFGF; PeproTech), and 20 ng/mL epidermal growth factor (EGF; PeproTech). The suspension was filtered through a 40 μm cell sieve (Falcon; BD Biosciences). Cells were plated into a 96-well plate in the presence of vehicle, GPR40 ligands GW9508 (10 or 50 μM), TUG905 (10 μM), and the antagonist GW1100 (10 μM; Tocris Bioscience) and incubated in a humidified incubator with a 5% CO_2_ atmosphere for 13 days to allow neurosphere growth and counting.

#### Postnatal neurosphere culture

Postnatal day 1 pups were euthanized, their brains immediately removed, and the hypothalamus microdissected. Tissue fragments were successively dissociated with a Pasteur pipette in PBS with 5.5 mM glucose, 100 U/mL penicillin, and 100 mg/mL streptomycin. Cells were suspended in 5 mL of proliferation media: Dulbecco’s modified Eagle’s medium (DMEM)-F12/Glutamax (Gibco) supplemented with growth factors (10 ng/mL bFGF and 10 ng/mL EGF, 100 U/mL penicillin, 100 mg/mL streptomycin, and 1% B27 supplement). The floating neurospheres were allowed to grow in uncoated 25 cm^2^ flasks incubated in a humidified incubator with a 5% CO_2_ atmosphere. On culture day 7, neurospheres were collected by centrifugation, dissociated, and plated, in fresh proliferation medium, onto Poly-D-Lysine (PDL; Sigma P1024)-coated 12 well culture plates for RNA extraction or glass coverslips for immunocytochemistry. After 5 days, or once the monolayer reached confluence, cell differentiation was induced by switching proliferation medium for media without growth factors for 7 or 18 days^33^. Cells were differentiated in the presence of 10 μM TUG905, BDNF (10 ng/mL; Sigma), IL-6 (2 ng/mL; Sigma), or SB 203,580 (0.1 μM Sigma) individually or in combination.

#### Neuro2a cell culture

The neuroblastoma Neuro2a cell line (ATCC CCL-131) was maintained in DMEM (Gibco) containing 4,500 mg/L glucose, 4 mM L-glutamine, 100 units/mL of penicillin, 100 μg/mL of streptomycin, and 10% fetal bovine serum. Incubation conditions were 37 °C in 5% CO_2_/humidified air. Neuro2 cells were plated in 6-well plates and after 24 h incubated with vehicle, 10 μM GW9508, or 10 μM TUG905 for 1 to 24 h and subsequently collected for mRNA and protein analysis.

### RNA extraction and quantitative real-time PCR

RNA samples were prepared using TRIzol (Invitrogen) according to the manufacturer’s recommendations. Spectrophotometry was employed for RNA quantification. For each sample, 2 μg of RNA was employed for the synthesis of complementary DNA (cDNA) using the High Capacity cDNA Reverse Transcription Synthesis kit (Applied Biosystems) according to the manufacturer’s recommendations. Real-time PCR reactions were run using the TaqMan system (Applied Biosystems). Primers used were GLAST (Mm00600697_m1); GLUT1 (Mm00441480_m1); FGF10 (Mm00433275_m1); IGFBP2 (Mm00805581); DCX (Mm00438400_m1); GFAP (Mm01253033_m1), AGRP (Mm00475829_g1); NPY (Mm00445771_m1); NEUROD1 (Mm01946604_s1); SOX2 (Mm03053810_s1); HCRT (Mm01964030_s1); POMC (Mm00435874_m1); GPR40 (Mm00809442_s1) SOX11 (Mm01281943_s1); Nestin (Mm00450205_m1); RAX (Mm01258704_m1); and BDNF (Mm01334043_m1). Analyses were run using 4 μL (10 ng/μL) cDNA, 0.625 μL primer/probe solution, 1.625 μL H_2_O, and 6.25 μL 2X TaqMan Universal MasterMix. GAPDH (Mm99999915_g1) was employed as a reference gene. Gene expression was obtained using the SDS System 7,500 software (Applied Biosystems).

### Western blot

Cell lysates were prepared, and protein extracts were incubated for 5 min at 95 °C with 4 × Laemmli sample buffer (1 mM sodium phosphate, pH 7.8, 0.1% bromophenol blue, 50% glycerol, 10% sodium dodecyl sulfate [SDS], and 2% β-mercaptoethanol). Electrotransfer of proteins to nitrocellulose membranes (Bio-Rad) was performed in a Trans-Blot SD Semi-Dry Transfer Cell (Bio-Rad) for 1 h at 15 V (constant) in buffer containing methanol and SDS. Following transfer, the membranes were blocked in 3% bovine serum albumin (BSA) solution in TBST (1 × TBS and 0.1% Tween 20) for 2 h, washed tree times 1 with TBST, and incubated with primary antibodies: rabbit anti-P-Akt (sc7985), rabbit anti-Akt (sc8312), mouse anti-P-ERK (8,149-2), or rabbit anti-P-P38 (sc7975) overnight at 4 °C. Horseradish peroxidase (HRP)-coupled secondary antibodies were used for detection of the chemiluminescence, and visualization was achieved by exposure to an Image Quant LAS4000 (GE Healthcare, Life Sciences), using mouse anti- α-tubulin (Sigma, T5168) as loading control. Scanned images were saved at high resolution (300 dpi) and bands were quantified by densitometry (UN-SCAN-IT gel analysis software). Results are expressed in relative fold change compared to control (vehicle 1 h). The uncut gel images are presented in Supplementary Data (Fig. S1).

### Immunocytochemistry

Cover slips containing differentiated neurospheres were fixed with 4% PFA in 0.1 M PBS for 10 min at RT. After washing with PBS, the cells were incubated in blocking solution (10% normal donkey serum in 0.1 M PBS containing 0.2% Triton X-100) for 1 h at RT. The cells were then incubated in fresh blocking solution (3% normal donkey serum) containing rabbit anti-DCX (1:200; Cell Signaling 4,604), rabbit anti-MAP2 (1:200; ab32454), or anti-GFAP Cy3 (1:2000; ab49874) overnight at 4 °C. The cells were washed three times with PBS and incubated in blocking solution containing donkey anti-rabbit FITC (1:500; ab6798) for 1 h at RT, followed by DAPI for 10 min at RT. Following another three PBS washes, the slides were mounted using fluorescence mounting medium before image capturing on fluorescence microscopy (Olympus BX41). The results of immunopositive cells represent the average of 2–3 cover slips per experimental replicate (different hypothalamic NPC culture preparations), where 4–5 fields were imaged per cover slip and averaged. The number of immunopositive cells was quantified per image using the ImageJ software and are expressed as percentage relative to control.

### Statistical analysis

Data were analyzed using GraphPad Prism and R. The statistical analyses were carried out using unpaired two-tailed Student’s t-test or one-way analysis of variance (ANOVA) when appropriate. Post hoc comparisons were performed using Tukey’s test. Data are presented as means ± standard error of the mean (SEM). A *p* value ≤ 0.05 was considered to be statistically significant. Full results of statistical tests are available in the Supplementary Table 1.

## Supplementary information

Supplementary file1
