# Supplementary material for: Activation of GPR40 induces hypothalamic neurogenesis through p38- and BDNF-dependent mechanisms
Source: Sci Rep. 2020 Jul 6;10:11047. doi: 10.1038/s41598-020-68110-2 (PMC7338363; doi:10.1038/s41598-020-68110-2)
Supplement: Supplementary file 1 — Supplementary file1 [file 41598_2020_68110_MOESM1_ESM.pdf]

Supplementary File

# Activation of GPR40 induces hypothalamic neurogenesis through p38- and BDNF-dependent mechanisms

Daiane F. Engel[1], Vanessa C. D. Bobbo[1], Carina S. Solon[1], Guilherme A. Nogueira[1], Alexandre Moura-Assis[1], Natalia F. Mendes[1], Ariane M. Zanesco[1], Athanasios Papangelis[2], Trond Ulven[2], Licio A. Velloso[1,*]

**FIGURE S1.** The uncut gel images of phospho-ERK, phospo-Akt, total Akt, phospho-P38 and alpha-tubulin immunocontent.

**SUPPLEMENTARY TABLE 1**. Statistical analysis for all parameters measured using a one-way ANOVA or t-test in the study.

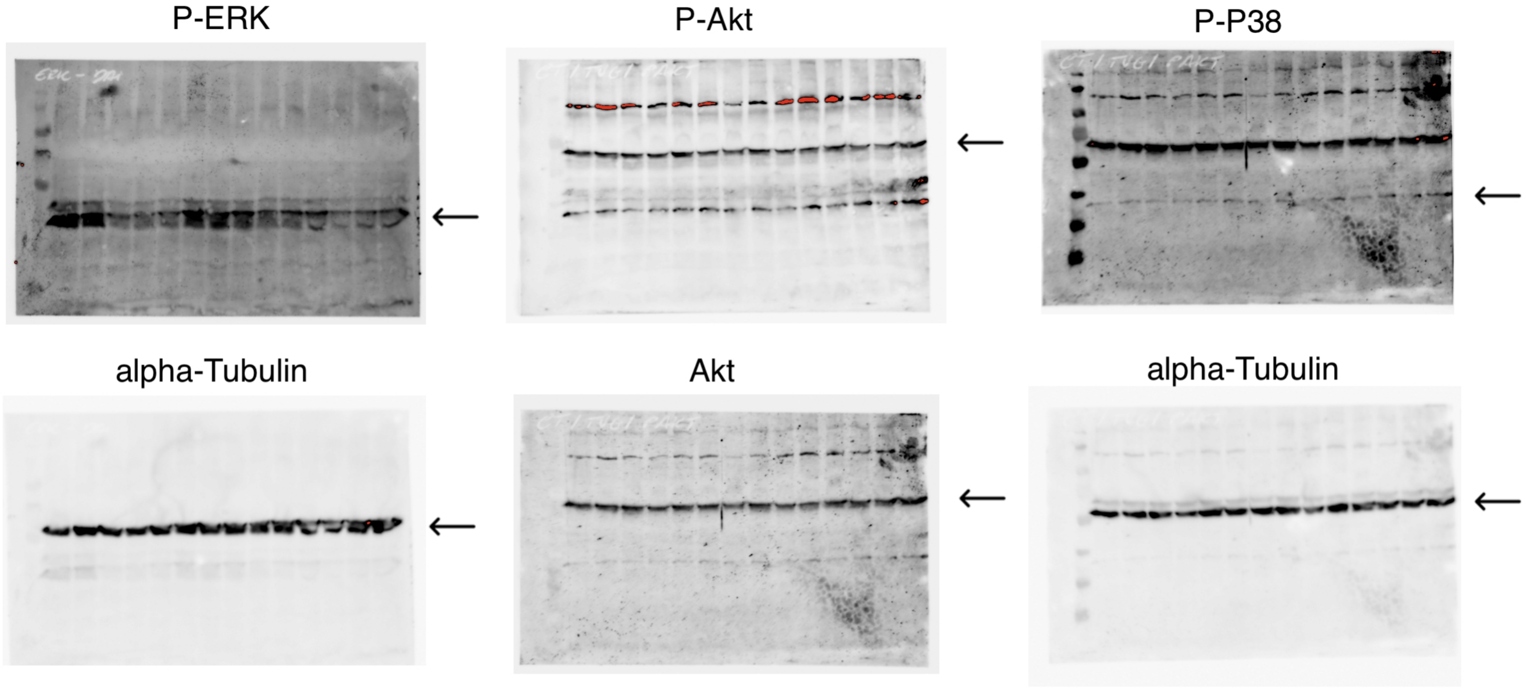
P-ERK
P-Akt
P-P38
alpha-Tubulin
Akt
alpha-Tubulin

| Figure | Analysis |
|---|---|
| **Fig 1C - HVZ** | one tailed; *t* = 1.98, df = 13; *p* = 0.035 |
| **Fig 1C - PA** | one tailed; *t* = 1.65, df = 13; *p* = 0.06 |
| **Fig 1D - HVZ** | one tailed; *t* = 2.24, df = 9; *p* = 0.026 |
| **Fig 1D - PA** | one tailed; *t* = 1.84, df = 9; *p* = 0.049 |
| **Fig 1H** | one way ANOVA [F (4, 12) = 60.30; *p* < 0.001 |
| | |
| **Fig 2C** | one way ANOVA [F (5, 18) = 0. 69; *p* = 0.64 |
| **Fig 2D** | one way ANOVA [F (5, 18) = 3. 16; *p* = 0.03 |
| **Fig 2E** | one way ANOVA [F (5, 16) = 11. 07; *p* < 0.001 |
| **Fig 2F** | one way ANOVA [F (5, 18) = 0. 61; *p* = 0.70 |
| **Fig 2G** | one way ANOVA [F (5, 18) = 0. 56; *p* = 0.73 |
| **Fig 2H** | one way ANOVA [F (5, 16) = 0. 98; *p* = 0.46 |
| **Fig 2I** | one way ANOVA [F (5, 18) = 2.06; *p* = 0.12 |
| **Fig 2J** | one way ANOVA [F (5, 18) = 1.15; *p* = 0.37 |
| **Fig 2K** | one way ANOVA [F (5, 12) = 7.58; *p* = 0.002 |
| | |
| **Fig 3B 2h** | Two tailed; *t* = 14.30, df = 4; *p* < 0.0001 |
| **Fig 3B 4h** | Two tailed; *t* = 0.98 df = 3; *p* = 0.40 |
| **Fig 3B 8h** | Two tailed; *t* = 1.90 df = 3; *p* = 0.15 |
| **Fig 3B 24h** | Two tailed; *t* = 0.75 df = 4; *p* = 0.49 |
| **Fig 3C 2h** | Two tailed; *t* = 0.73 df = 4; *p* = 0.52 |
| **Fig 3C 4h** | Two tailed; *t* = 1.50 df = 4; *p* = 0.21 |
| **Fig 3C 8h** | Two tailed; *t* = 1.14 df = 4; *p* = 0.32 |
| **Fig 3C 24h** | Two tailed; *t* = 2.86 df = 4; *p* = 0.046 |
| **Fig 3D 2h** | Two tailed; *t* = 3.89 df = 6; *p* = 0.0081 |
| **Fig 3D 4h** | Two tailed; *t* = 5.09 df = 6; *p* = 0.0022 |
| **Fig 3D 8h** | Two tailed; *t* = 0.63 df = 6; *p* = 0.55 |
| **Fig 3D 24h** | Two tailed; *t* = 0.41 df = 6; *p* = 0.70 |
| **Fig 3E 2h** | Two tailed; *t* = 1.13 df = 6; *p* = 0.30 |
| **Fig 3E 4h** | Two tailed; *t* = 2.64 df = 6; *p* = 0.038 |
| **Fig 3E 8h** | Two tailed; *t* = 0.25 df = 4; *p* = 0.82 |
| **Fig 3E 24h** | Two tailed; *t* = 2.66 df = 6; *p* = 0.037 |
| **Fig 3F 1h** | Two tailed; *t* = 0.69 df = 3; *p* = 0.54 |
| **Fig 3F 4h** | Two tailed; *t* = 0.30 df = 4; *p* = 0.78 |
| **Fig 3G 1h** | Two tailed; *t* = 2.58 df = 3; *p* = 0.08 |
| **Fig 3G 4h** | Two tailed; *t* = 2.06 df = 4; *p* = 0.11 |
| **Fig 3H 1h** | Two tailed; *t* = 1.10 df = 3; *p* = 0.35 |
| **Fig 3H 4h** | Two tailed; *t* = 2.59 df = 4; *p* = 0.03 |
| | |
| **Fig 4B** | one way ANOVA [F (2, 22) = 5.77; *p* = 0.0046 |
| **Fig 4C** | one way ANOVA [F (3, 8) = 10.45; *p* = 0.0038 |
| **Fig 4D** | one way ANOVA [F (3, 27) = 2.35; *p* = 0.09 |
| **Fig 4E** | one way ANOVA [F (3, 28) = 43.17; *p* < 0.0001 |
| **Fig 4F** | one way ANOVA [F (3, 28) = 0.28; *p* = 0.84 |
| **Fig 4G** | one way ANOVA [F (3, 8) = 7.13; *p* = 0.01 |
| **Fig 4I** | one way ANOVA [F (3, 15) = 4.89; *p* = 0.014 |

**Fig 4J** one way ANOVA [F (3, 14) = 1.40; *p* = 0.28
